# Gender aspects suggestive of gastroparesis in patients with diabetes mellitus: a cross-sectional survey

**DOI:** 10.1186/1471-230X-14-34

**Published:** 2014-02-19

**Authors:** Ram Dickman, Julio Wainstein, Marek Glezerman, Yaron Niv, Mona Boaz

**Affiliations:** 1 Departments of Gastroenterology, Rabin Medical Center, Beilinson Campus, Petach Tikva, Israel; 2 Diabetes Unit, Tel Aviv University, Tel Aviv, Israel; 3 Epidemiology Unit, The Edith Wolfson Medical Center, Tel Aviv University, Holon, Tel Aviv, Israel; 4 Research Center for Gender Medicine, Tel Aviv University, Tel Aviv, Israel; 5 Sackler Faculty of Medicine, Tel Aviv University, Tel Aviv, Israel

**Keywords:** Diabetes mellitus, Diabetic gastroparesis

## Abstract

**Background:**

It is suggested that symptoms related to gastroparesis are more common in female than in male patients with type 2 diabetes mellitus (T2DM). The association between sex and prevalence of symptoms suggestive of gastroparesis among patients with T2DM in Israel has not been reported. The aim of this study was to describe the associations between sex, clinical characteristics, type, severity and prevalence of dyspeptic symptoms in a large population of patients with T2DM in Israel.

**Methods:**

All patients completed a demographic questionnaire and the Gastroparesis Cardinal Symptom Index (GCSI). Data regarding disease duration, medications, complications, recent blood glucose and HbA1c levels were also collected. In this nested case–control study, 173 female and 209 male patients were identified from within a cross-sectional survey of 382 patients with T2DM. Logistic and general linear modeling was used to assess associations between sex, clinical data, and the presence (type and number) of symptoms.

**Results:**

Compared to males, female patients with T2DM had a higher body mass index (BMI) (31.9 vs. 29.2; *P* = 0.001) and HbA1c levels (7.9 vs. 7.5; *P* = 0.04). A larger proportion of males suffered from peripheral vascular disease (*P* = 0.02) and ischemic heart disease (*P* = 0.001). Other disease characteristics did not differ between the sexes. The prevalence of nausea (*P* = 0.001), early satiety (*P* = 0.005), loss of appetite (*P* = 0.002), or presence of any cardinal symptom (*P* = 0.001) was significantly higher among females. Severity of most cardinal symptoms was also higher in females. The presence of at least one cardinal symptom was more likely among obese females with longer disease duration and poor glycemic control.

**Conclusions:**

Prevalence and severity of symptoms suggestive of gastroparesis is particularly high among obese females with long standing and poorly controlled T2DM.

## Background

Gastroparesis is characterized by an abnormal gastric emptying rate (GER) associated with symptoms, such as nausea, vomiting, postprandial fullness, early satiety, and bloating [1,2]. Diabetic gastroparesis (DG) affects patients with long-standing diabetes mellitus (DM) usually complicated by retinopathy, neuropathy, and nephropathy [1]. DG may cause severe nutritional deficits, and is associated with higher morbidity and mortality due to difficulties in achieving glucose control [1,3]. Early diagnosis of DG is needed because therapy by strict glucose control, diet interventions and prokinetics often leads to clinical improvement [4].

There is no perfect biomarker for dyspeptic symptoms suggestive of gastric dysmotility, such as DG. In fact, GER was found to be surprisingly normal in some dyspeptic patients with long- term DM, or delayed in asymptomatic patients with type 2 DM (T2DM) [5]. Nevertheless, the current definition for DG still relies on demonstrating delayed gastric emptying by gastric scintigraphy in symptomatic patients [1,4]. Gastric scintigraphy requires specialized expensive equipment and imposes a low but measurable radiation exposure. Thus, in view of the above, it is reasonable to pre-select diabetic patients for a further evaluation by gastric scintigraphy based on the presence of cardinal symptoms suggestive of DG [2]. Postprandial fullness, early satiety, bloating, nausea and vomiting have been reported to be more prevalent in diabetic patients than in the general population. In a gastric scintigraphy study of 101 patients with DM, 83% reported upper gastrointestinal symptoms [6]. Conversely, only 7-15% of subjects in the general population reported dyspeptic symptoms [7].

The underlying mechanisms for DG include autonomic neuropathy, enteric neuropathy, abnormalities of interstitial cells of Cajal, poor (i.e. acute fluctuations) glucose control, use of incretin-based medications, and psychosomatic factors [1]. Females generally have a slower solid and liquid GER than males [8-10]. The underlying mechanism for this phenomenon is not fully understood and may be related to estrogen levels. Indeed, during the ovulatory period and during pregnancy, females tend to have reduced peristalsis and increased incidence of intractable constipation.

The aim of the present study was to further investigate the associations between sex, clinical data, type, severity and prevalence of cardinal symptoms suggestive of gastroparesis in a large population of patients with T2DM in Israel.

## Methods

A cross-sectional survey of 382 consecutive patients with T2DM, treated in community outpatient clinics, was performed to estimate the prevalence of gastroparesis. All patients completed a demographic and lifestyle questionnaire as well as the validated Hebrew translation of the Gastroparesis Cardinal Symptom Index (GCSI) [11]. Height and weight were recorded and body mass index (BMI) was calculated as weight [kg)/(height (m)^2^]. Disease duration, prescribed medications, comorbidities, diabetes complications, most recent blood glucose and HbA1c levels were extracted from the patients’ medical records. This community center study was conducted in accordance with the principles of the Declaration of Helsinki and Good Clinical Practice (GCP) and was approved by the Human Subjects Protection Program of The Edith Wolfson Medical Center. All participants provided written informed consent before enrollment.

### Symptom assessment

Patients were asked to rate symptoms suggestive of gastroparesis. The GCSI consists of three subscales of the Patient Assessment of Upper Gastrointestinal Disorders-Symptom Severity Index PAGI-SYM, selected to measure important symptoms related to gastroparesis. Symptoms include nausea/vomiting, postprandial fullness/early satiety, and bloating [2]. The nausea/vomiting subscale includes the following three items: Nausea, retching, and vomiting. The postprandial fullness/early satiety subscale is comprised of the following four items: Stomach fullness, inability to finish a normal-sized meal, feeling excessively full after meals, and loss of appetite. The bloating subscale is comprised of the following two items: Bloating, and visibly larger stomach or belly after meals. Overall, the GCSI comprises 9 questions and each question is rated by the responder according to severity of symptoms, i.e. from 0 to 5 (0 = no symptoms to 5 = severe symptoms). The total GCSI score was categorized as “severe” (GCSI > 27) or “mild” (GCSI ≤ 27).

### Definitions

“Cardinal symptoms” included nausea, retching, vomiting, difficulty completing a normal meal, early satiety, post-prandial fullness, loss of appetite, bloating, and visible postprandial abdominal distension. “Obese” was defined as BMI ≥ 30 kg/m^2^.

### Sample size and study power

The survey cohort included 382 patients with T2DM. This sample size provided 80% power to detect a true, by-sex relative difference in the presence of any cardinal symptom of approximately 15% (assuming a rate of 60% in males and 70% in females) regarding the presence of any cardinal symptom. This estimate is based on the chi square test and assumes a two-sided alpha of 0.05.

### Data analysis

Analysis of data was performed with the SPSS 21.0 statistical analysis software (IBM Inc., USA). Distributions of continuous variables were assessed for normality using the Kolmogorov-Smirnov test (cutoff at *P* < 0.01). Normally distributed continuous variables, such as age, height and weight were described as mean ± standard deviation (m ± SD). The t-test for independent samples or the Mann–Whitney U was used to compare continuous variables by sex. For categorical variables, such as comorbidities and medications, we used frequency distributions, presented as frequency (%). The chi square test (Fisher's exact as needed) was used to assess associations between categorical variables and sex. The presence of any cardinal symptom was modeled using logistic regression analysis. Odds ratios (ORs) were estimated with 95% confidence intervals (CIs). All tests were two-sided and considered significant at *P* <0.05.

## Results

In this study, 173 female and 209 male patients were included in the cross-sectional survey of patients with T2DM.

Table 1 describes the clinical characteristics of the study participants. Compared to males, a greater proportion of female patients was obese and had poorer glycemic control. A larger proportion of males had peripheral vascular disease and ischemic heart disease. Other by-sex clinical differences were not significant. Self-reported adherence to dietary counseling was similar but the proportion of patients reporting regular exercise was lower among females.

**Table 1:** Disease characteristics of 382 T2DM patients compared by gender

	**Females N=173**	**Males N=173**	P value**
Age (years)*	63.5±10.1	62.7±11.5	0.48
BMI (kg/cm^2^)*	31.9±5.2	29.2±9.7	0.001
Obesity (%)	59.0	27.3	<0.0001
Diabetes duration (years)*	14.0±11.9	11.9±8.6	0.05
Fasting blood glucose (mg/dl) *	154.5±64.1	146.1±57.2	0.18
HbA1c (%)*	7.9±1.4	7.5±1.6	0.04
Diabetes complications:			
Peripheral vascular disease (%)	12.8	21.5	0.02
Ischemic heart disease (%)	32.0	49.3	0.001
Hypertension (%)	84.2	82.3	0.62
Cerebral vascular accident	9.9	8.1	0.55
Dyslipidemia (%)	86.7	79.9	0.07
Diabetes medications:			
Metformin (%)	66.5	60.3	0.21
Sulfonylureas (%)	53.2	56.0	0.58
Acarbose (%)	9.2	11.1	0.56
Rosiglitazone (%)	6.9	12.9	0.05
Exenatide (%)	5.2	4.8	0.85
Insulin (%)	49.1	44.0	0.31
Other medications:			
Statins (%)	82.6	79.9	0.51
Alfa blockers (%)	14.0	21.6	0.53
ACE inhibitors (%)	79.7	78.0	0.69
Calcium channel blockers (%)	34.9	28.4	0.17
Beta blockers (%)	47.1	52.2	0.32
Diuretics (%)	36.0	36.8	0.87
Nitrates (%)	13.4	14.8	0.68
Lifestyle characteristics:			
Exercise (%)	53.8	68.9	0.02
Adherence to dietary counseling (%)	86.0	87.1	0.76

Values are expressed as percentage.

*Mean ± standard deviation; Obesity = BMI ≥ 30 kg/cm^2^.

**P value is for across-group comparison; post hoc testing was conducted for significant findings.

### Prevalence and severity of symptoms by gender

The prevalence and severity of the reported cardinal symptoms were compared by sex in Tables 2 and 3, respectively. Females patients reported significantly more nausea (*P* = 0.001), early satiety (*P* = 0.001), feeling excessively full after meals (*P* = 0.005), and loss of appetite (*P* = 0.002). Sex differences in retching, vomiting, stomach fullness, bloating, and visibly larger stomach or belly after meals were not detected (Table 3). The reported severity of most symptoms (except for retching and vomiting) was also significantly higher among females (Table 3). The presence of at least one cardinal symptom was more likely among female patients who reported (of more) other symptoms as well.

**Table 2:** Subject’s prevalence of symptoms suggestive of gastroparesis compared by gender (%)

	**Females N = 173**	**Males N = 209**	P value**
Nausea (%)	31.8	31.8	0.001
Retching (%)	9.8	9.8	0.19
Vomiting (%)	6.9	6.9	0.26
Stomach fullness (%)	45.7	45.7	0.17
Early satiety (%)	36.7	36.7	0.001
Feeling excessively full after meals (%)	52.6	52.6	0.005
Loss of appetite (%)	23.1	23.1	0.002
Bloating (%)	53.2	53.2	0.11
Stomach or belly visibly larger (%)	57.8	57.8	0.79
Any symptom (%)	82.1	82.1	0.003
No. of symptoms*	3.4±2.5	3.4 ± 2.5	0.001

*Mean ± standard deviation.

**P value is for across-group comparison; post hoc testing was conducted for significant findings.

**Table 3:** Subject’s mean severity score of symptoms suggestive of gastroparesis compared by gender

	**Females N = 173**	**Males N = 209**	***P *** value*
Nausea	0.75	0.38	0.002
Retching	0.18	0.11	0.20
Vomiting	0.13	0.09	0.44
Stomach fullness	1.32	1.00	0.04
Early satiety	0.89	0.47	0.001
Feeling excessively full after meals	1.57	1.06	0.002
Loss of appetite	0.53	0.24	0.003
Bloating	1.60	1.02	0.013
Stomach or belly visibly larger	1.78	1.32	0.009

Values are expressed as mean ± standard deviation.

*P value is for across-group comparison; post hoc testing was conducted for significant findings.

### Associations between symptoms and disease characteristics

A logistic regression model (Table 4) was developed to assess factors that predict symptoms. Sex, age, diabetes duration, HbA1c levels and BMI were included in the model. The model was significant (*P* < 0.0001) and correctly categorized 75% of study participants as having at least one gastroparesis symptom. Specifically, female sex increased symptom risk by 83.8%: OR 1.838, 95% CI 1.099-3.074, *P* = 0.02.

**Table 4:** Logistic regression of any symptom suggestive of gastroparesis

	**OR**	**95% CI**	***P *** **value**
BMI	1.026	0.981-1.074	0.277
Female sex	1.838	1.099-3.074	0.020
Age	1.013	0.990-1.069	0.209
HbA1c	1.093	0.922-1.297	0.306
Disease duration	1.026	0.998-1.056	0.073

*BMI* body mass index.

## Discussion

In the present study, female patients with T2DM reported more dyspeptic symptoms that may suggest gastroparesis. We also found that the symptom severity scores were higher among females. Other studies that evaluated gastric emptying in patients with T2DM found that females tend to have a slower GER [8,9,12,13]. Furthermore, in an ongoing research study by our group, a significantly greater proportion of age-matched female patients without T2DM (control group) were found to have a slower GER for solids than females with T2DM. This finding too may suggest a sex-relative effect on GER as observed in this study. The underlying mechanism for this phenomenon is not fully understood and seems multifactorial. In humans, the slower gastric motility in females may be attributable to estrogen levels. Reduction in sex steroid hormones may down regulate NOS-mediated gastric motility leading to gastroparesis in diabetic females [10]. However, in our study, most of the females with T2DM were in their 6^th^ decade of life indicating low, rather than normal or high estrogen levels. Unfortunately, we have insufficient information related to hormonal replacement therapy (HRT), that some of our female patients might have received. Consequently, a conclusion regarding the probable effect of estrogen (based on reports of HRT prescriptions) could not be drawn.

In the present study, most females with T2DM were obese. According to the results of a recent study, it was suggested that obesity could play a significant role in estrogen receptor gene expression [14]. It is the adipose tissue of obese vs non-obese females that secretes more biologically active estrogen. Thus, we may only speculate that in our obese female patients with T2DM, biologically active estrogen and estrogen receptor gene expression were higher than in non-obese patients. Finally, even if there are sex differences in gastric emptying, perhaps mediated by estrogen levels, the generally low prevalence of delayed emptying in symptomatic individuals makes it highly unlikely that a sex-dependent phenomenon is the only mechanism that affects gastric emptying.

Our findings must be considered in light of the study’s limitations. Cardinal symptoms were suggestive of gastroparesis, but this condition was not objectively diagnosed by gastric scintigraphy. Rey *et al.*[15] recently assessed the possible presence of gastroparesis in the general population. Instead of dichotomizing based on mere symptom presence as performed in our study, these authors examined the presence of documented gastroparesis in a clinic sample, and correlated symptom scores with presence of gastroparesis. While questionable (delayed gastric emptying poorly correlates with symptoms), they used these data to predict the presence of gastroparesis based on weighted symptom severity scores. Not surprisingly, the prevalence was slightly lower, as they dealt with a clinic population and had a higher threshold for case identification. Nevertheless, Rey *et al.* concluded that since the prevalence of diagnosed gastroparesis is low, many subjects with gastroparesis may remain undiagnosed. As the data presented in our study do not include a sample with clinical and gastric emptying data, we can only discuss symptoms suggestive of gastroparesis rather than diagnosed gastroparesis. As mentioned, gastroparesis is defined by demonstrating delayed gastric emptying in a symptomatic patient [4]. However, as the correlation between subjective and objective evidence for gastroparesis is poor in patients with DM, we believe that the absence of an imperfect biomarker, such as a gastric scintigraphy, may not be a real weakness.

Though symptoms may be attributed to conditions other than gastroparesis, such as functional gastrointestinal disorders, identifying the elevated prevalence of these symptoms among diabetic patients is important for the clinical practitioner. Firstly, knowledge regarding the prevalence of a condition in a given population permits the clinician to consider prior probability when encountering these symptoms in a specific individual. Secondly, knowing that symptoms are common within certain patient groups can serve to reassure the anxious patient.

Another limitation is that our study was conducted in a convenience sample of individuals, and not in a probability sample of all patients with DM. This makes generalization to the target population challenging. Nevertheless, the size and source (a large community-based clinic) of the study population and its composition suggests a robustness of findings. An additional important weakness is related to the study criteria for illness definition that may be set too low. In fact, with a relatively minor symptom severity and no indicators of symptom duration, many of our patients may not fulfill current consensus criteria for functional dyspepsia. Furthermore, important confounders in our study may play a significant role in explaining the results. These include an older age and comorbid conditions with multiple treatments, such as calcium channel blockers (constipation and bloating), acarbose (bloating) and exenatide (nausea). Thus, we may not be dealing with a secondary consequence of DM, but rather the consequence of treating disease. Finally, we could not discuss the probable effect of estrogen level based on reports of HRT prescriptions as this information was unavailable for most of our females. Nevertheless, as most of the symptomatic females were obese, we may still speculate (based on our model) that estrogen level and receptor gene expression were higher in our female patients with T2DM.

The identification of obesity as a risk factor for gastroparesis symptoms in females with T2DM provides an opportunity for intervention, and suggests that weight loss may improve symptoms, especially in this patient population [16]. A controlled clinical trial designed to examine this intervention is the next logical step.

## Conclusions

The prevalence and severity of symptoms suggestive of gastroparesis among patients with T2DM is higher in females than in males. This association persists even after controlling for disease duration, age, HbA1c percentage and BMI. These findings suggest that when treating patients with T2DM, physicians may want to screen for gastric symptoms suggestive of gastroparesis using the GCSI. This seems particularly relevant for female patients.

## Abbreviations

BMI: Body mass index; CI: Confidence interval; DG: Diabetic gastroparesis; GCSI: Gastroparesis cardinal symptom index; GER: Gastric emptying rate; HRT: Hormonal replacement therapy; m ± SD: mean ± standard deviation; NOS: Nitric oxide; OR: Odds ratio; T2DM: Type 2 diabetes mellitus.

## Competing interest

The authors declare that they have no competing interests.

## Authors’ contributions

RD was responsible for the conception, design and conducting of the study and the drafting and approval of the manuscript version to be published. JW was responsible for the conception, design and conducting of the study and the drafting and approval of the manuscript version to be published. MG was responsible for interpretation of the data, drafting and approval of the manuscript version to be published. YN was responsible for the analysis, drafting and approval of the manuscript version to be published. MB was responsible for the statistical analysis and interpretation of the data and the drafting and approval of the manuscript version to be published.

## Pre-publication history

The pre-publication history for this paper can be accessed here:

http://www.biomedcentral.com/1471-230X/14/34/prepub
